# Supplementary material for: Evaluation of the Neutralizing Antibody STE90-C11 against SARS-CoV-2 Delta Infection and Its Recognition of Other Variants of Concerns
Source: Viruses. 2023 Oct 25;15(11):2153. doi: 10.3390/v15112153 (PMC10675157; doi:10.3390/v15112153)
Supplement: Supplementary file 1 [file viruses-15-02153-s001.zip › viruses-2653793-supplementary.pdf]

Supplementary Data

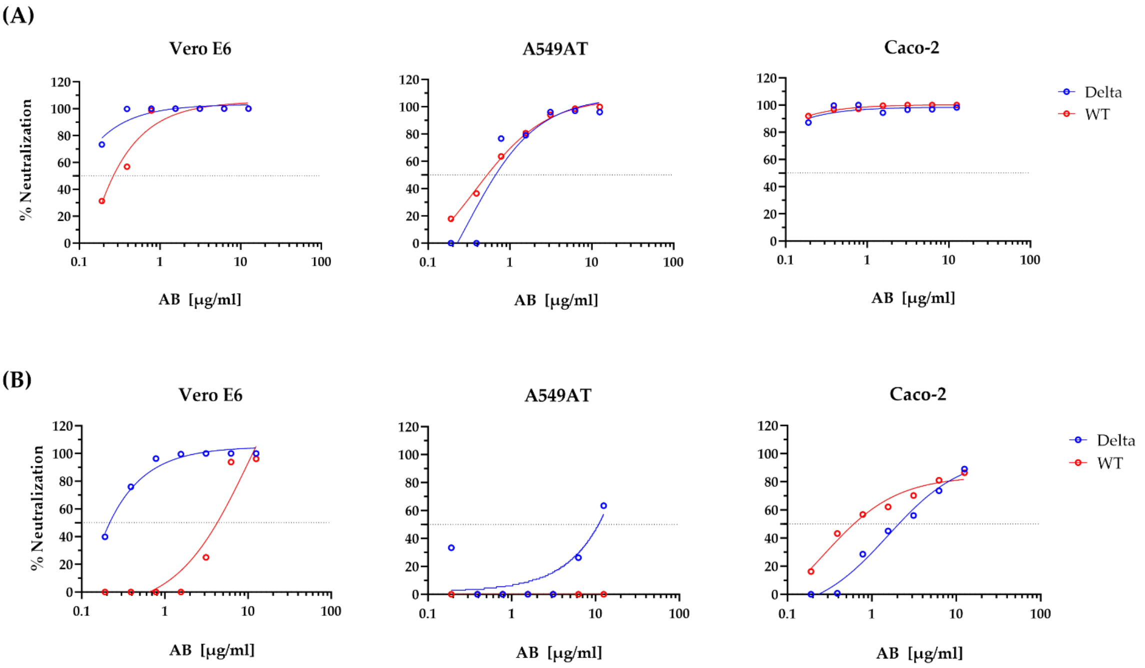

Supplementary Figure S1. Authentic SARS-CoV-2 neutralization titration by STE90-C11. STE90-C11 neutralization capacity was tested on different cell types at different concentrations. The percentage of neutralized extracellular (A) and intracellular (B) virions was assessed in different cell types. Lines represent nonlinear regression fit and data were shown as mean±SEM of one representative experiments with three technical replicates.

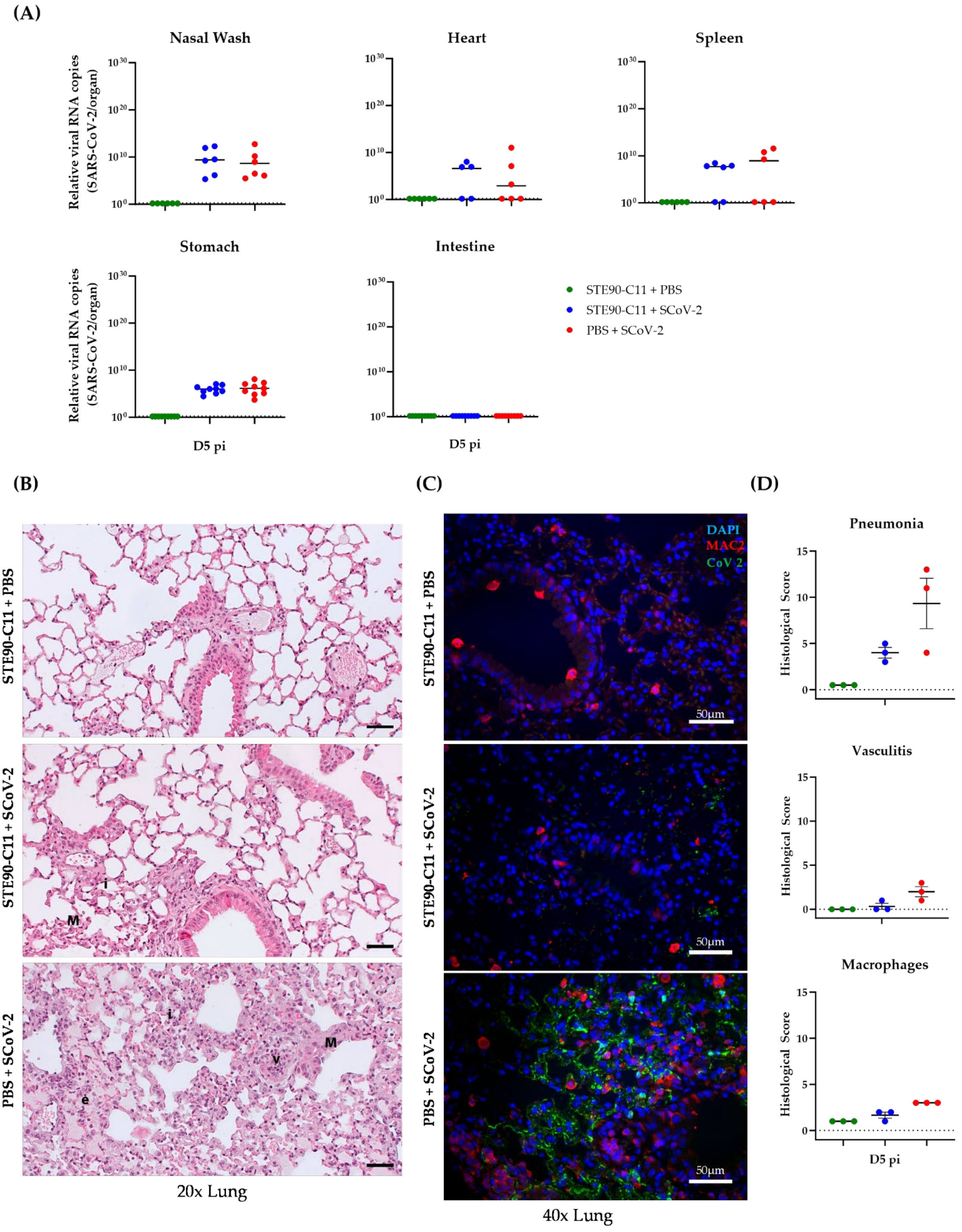

Supplementary Figure S2. STE90-C11 reduces inflammation in the lung tissue. K18-hACE 2 transgenic mice received 30mg/kg of STE90-C11 by intravenous injection one hour before intranasal inoculation with 2x10^3 PFU SARS-CoV-2 Delta strain in 20μL volume. Organs were collected at 5 days post-infection. (A) Viral RNA levels in different tissues were measured. The dotted line indicates the limit of detection of the assay. Pooled data (n=6-8 per group) from three independent experiments are shown. Each symbol is an individual mouse, and horizontal lines indicate the median of biological replicates. (B) Representative hematoxylin-eosin (HE) staining of lung tissue from PBS (top), STE90-C11 treated (middle), and only infected (bottom). I= interstitial pneumonia, V=vasculitis, E= alveolar edema and M=macrophages. (C) Representative images of immunohistochemically stained for the nucleocapsid CoV-1/2 (green), MAC2 (red), and DAPI (blue) in lung tissue of PBS (top), STE90-C11

treated (middle) and only infected (bottom). Scale bars, 50µm. (D) Histological score analysis for Pneumonia, Vasculitis, and Macrophages. Mean Values± SEM from 3 animals per group day 5 pi.

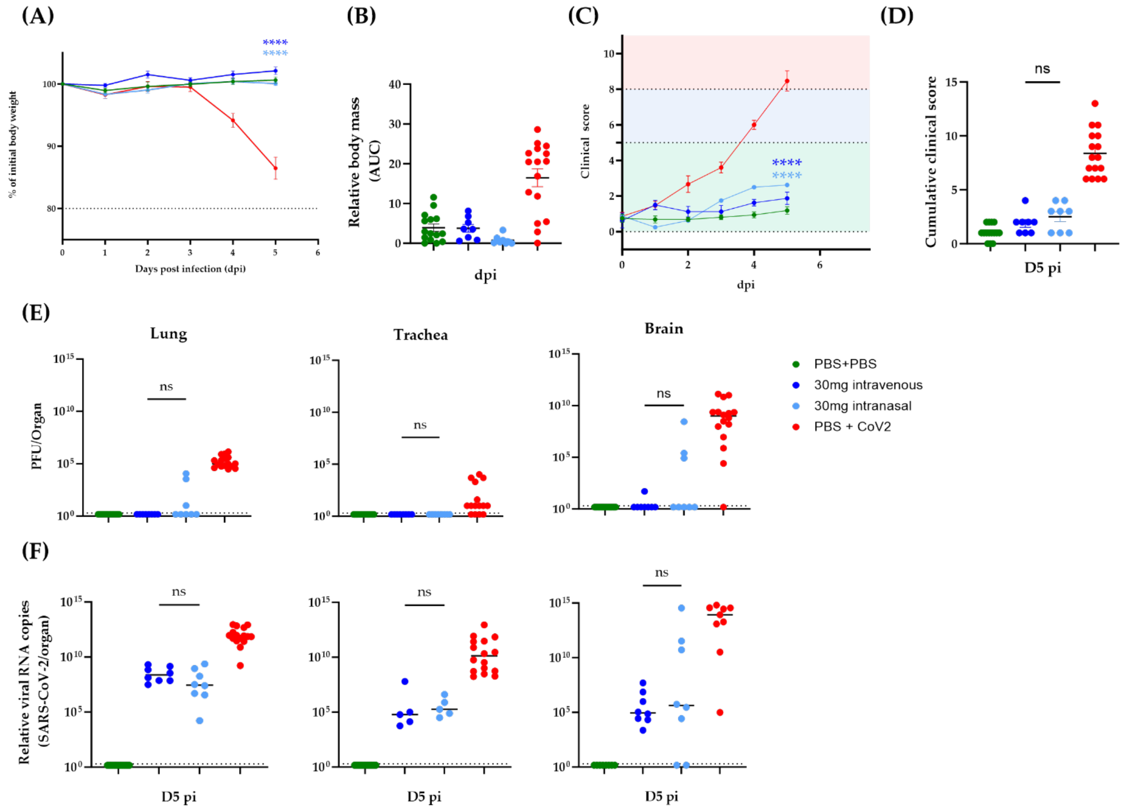

Supplementary Figure S3. Comparison of route application for STE90-C11. K18-hACE 2 transgenic mice received 30mg/kg of STE90-C11 or PBS by either intravenous (blue) or intranasal injection (light blue) two days upon infection with 2x10³ PFU SARS-CoV-2 Delta strain in 20µL volume intranasal. Organs were collected at 5 days post-infection. (A) Weight change after infection with SARS-CoV-2. (B) Cumulative relative mass reduction in SARS-CoV-2 infected and with mAb treated mice until D5 pi are shown as area under the curve (AUC). (C) Daily clinical scores upon SARS-CoV-2 infection and mAb treatment. The indicated thresholds represent the clinical severity of mice; low (green), moderate (blue), and severe (red, humane end-point). (D) Cumulative clinical score on D5 pi. (E) SARS-CoV-2 viral load at D5 pi in the lung (left), trachea (middle), and brain (left). The dotted lines indicate the limit of detection (LOD) for live virus titers. (F) Viral RNA levels in the lung, trachea, brain, and nasal wash were measured. The dotted line indicates the limit of detection of the assay. Pooled data from three independent experiments are shown. Each symbol is an individual mouse, and horizontal lines indicate the median of biological replicates. Statistical significance versus the route application was calculated using (D, E) Kruskal-Wallis test followed by Dunn´s post-analysis, and (F) Mann-Whitney test. ns= non-significant

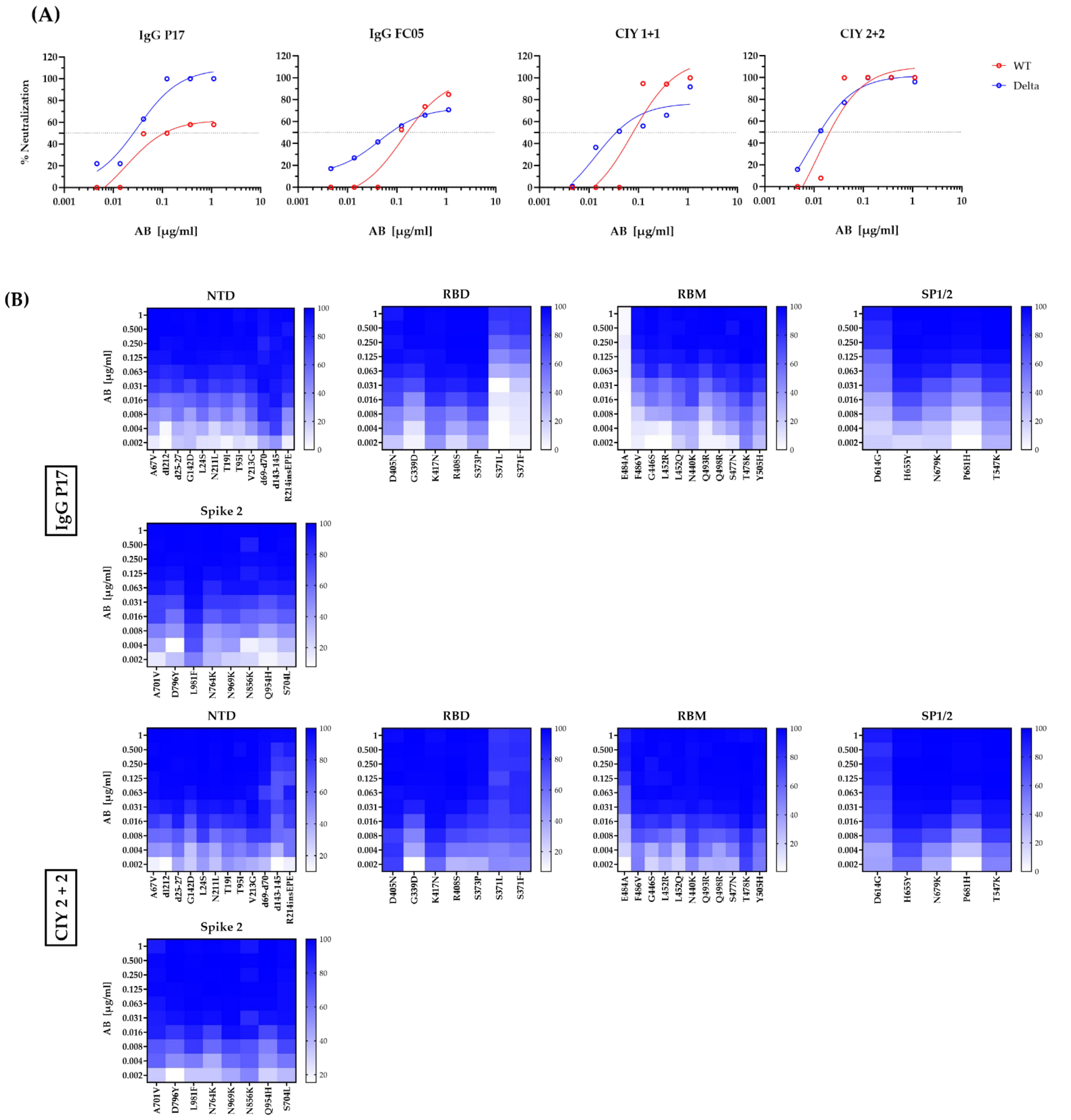

Supplementary Figure S4. The neutralization profile of the bispecific mAbs against authentic Wild-type and Delta viruses was represented as a percentage of neutralization (A) and single mutation mapping of IgG P17 and CIY 2+2 was represented as a heatmap.
